# Left Circumflex Artery Rupture with Left Atrial Tamponade and Functional Mitral Stenosis

**DOI:** 10.1155/2018/8676572

**Published:** 2018-02-12

**Authors:** Weiting Huang, Khaled Mohamed Emadeldin Moheb Hammad, Victor Tar Toong Chao, Khung Keong Yeo

**Affiliations:** ^1^National Heart Centre Singapore, Singapore; ^2^National Heart Institute, Cairo, Egypt

## Abstract

The growth in percutaneous transluminal devices has enabled operators to tackle more complex, native, and post-bypass surgery anatomy. However, complications such as coronary artery dissection, coronary perforation, retrograde aortic dissection, arrhythmias, and acute coronary syndrome still occur with resulting mortality rates of up to 4.2% in complex interventions. Perforation of the circumflex artery is of particular interest in view of its position and relation to the surrounding cardiac structures. This is a site of potential fluid collection, and as the left atrium is fixed to the parietal pericardium at the entry of the pulmonary veins, fluid in the oblique sinus can accumulate enough pressure to compress the left atrium and the coronary sinus. We present a case of left circumflex artery perforation which demonstrates the physiologic complications of coronary sinus and left atrial compression and the resultant functional mitral stenosis.

## 1. Case

**Figure 1:**
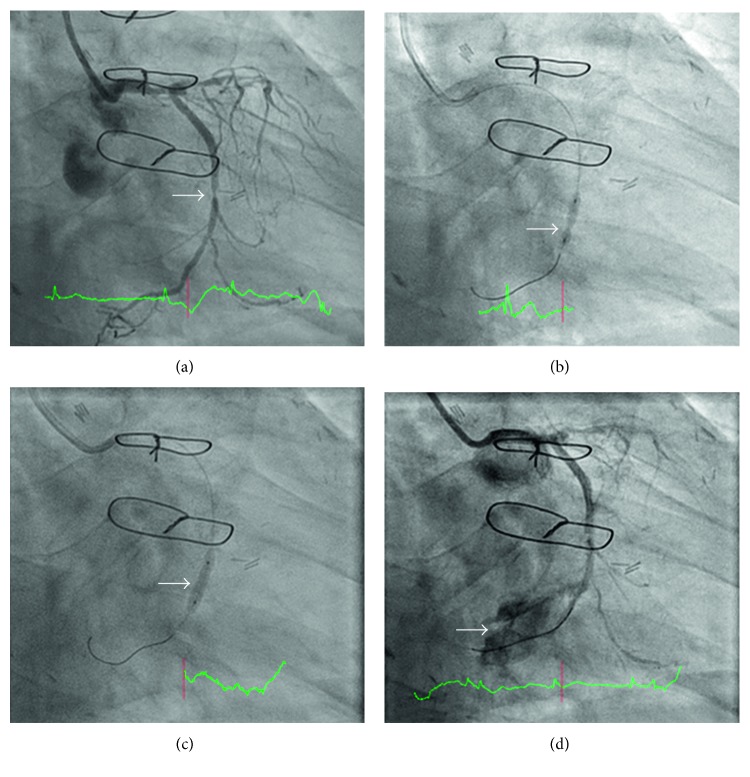
(a) Severe stenosis of the mid circumflex artery before intervention. The circumflex provides collaterals to the RCA. (b) Persistent “waisting” (stenosis) after dilating with a 2.5 mm compliant balloon. (c) Loss of waist after inflation of a 2.5 mm AngioSculpt balloon. (d) Ellis III perforation after dilation with a 2.5 mm scoring balloon.

A 70-year-old gentleman was admitted to the emergency room for unstable angina. He underwent coronary artery bypass grafting 6 years prior to current admission and also had persistent atrial fibrillation on rate control and anticoagulation. His ejection fraction was 65%. Coronary angiogram showed occlusion of all vein grafts but patent left internal mammary graft to left anterior descending artery with severe native multivessel disease. There was severe stenosis of the native left circumflex artery (Figure 1(a)); the proximal right coronary artery was completely occluded, and it was supplied distally by bridging collaterals and collaterals from the left circumflex artery. After extensive discussion with the patient, decision was made for percutaneous coronary intervention (PCI) of the native left circumflex artery. Serial balloon dilation to the mid circumflex artery was performed: first with a 2.5 mm × 15 mm balloon but a waist was noted (Figure 1(b)), followed by a noncompliant 2.5 mm × 12 mm balloon but a residual waist was still observed, and finally with a 2.5 mm × 10 mm scoring balloon (AngioSculpt, Spectranetics, Milwaukee, Wisconsin, USA) inflating up to 20 atmospheres (Figure 1(c)). The stenosis gave way, and repeat angiogram noted Ellis Class III perforation (Figure 1(d)).

The operators immediately inflated a 3.0 mm balloon to 8 atmospheres at the site of perforation followed by deployment of a 2.5 × 24 mm covered stent. However, repeat angiography still showed extravasation from perforation site and a second 2.5 × 12 mm covered stent was deployed, but there was continued extravasation. Protamine sulfate was given, and prolonged balloon inflation was then performed. Subsequent angiography showed that the circumflex artery was thrombosed.

Immediate bedside transthoracic echocardiogram showed very small 4 mm pericardial effusion. Pericardiocentesis was attempted but was unsuccessful. The ejection fraction was normal, and there were no other echocardiographic features of cardiac tamponade.

**Figure 2:**
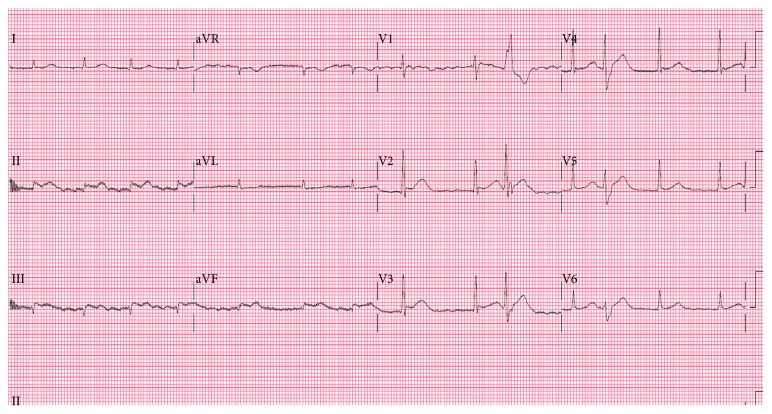
ECG showing ST elevation in inferior leads.

**Figure 3:**
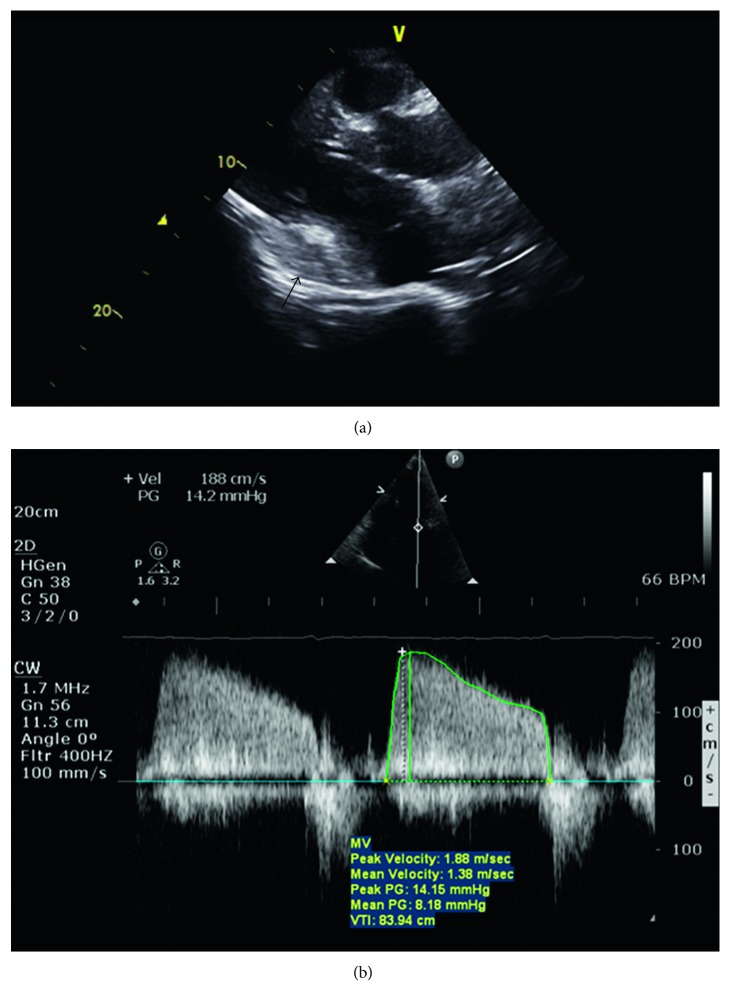
(a) Emergency transthoracic echocardiogram showing accumulation of hematoma around the coronary sinus and atrioventricular groove. (b) Pulse Doppler in the apical 4-chamber view across the mitral valve showing elevated mean pressure gradient of 8 mmHg, suggesting functional mitral stenosis secondary to compression of the left atrium and mitral valve apparatus.

The patient remained hemodynamically stable for about 20 minutes before developing progressive hypotension. The ECG showed fast atrial fibrillation with acute ST elevation in II, III and aVF (Figure 2). He was supported with inotropes but developed pulseless ventricular tachycardia requiring cardioversion and 10 minutes of cardiopulmonary resuscitation. Venoarterial extracorporeal membrane oxygenation (VA ECMO) was inserted for hemodynamic stabilization. Repeat assessment by echocardiogram showed no increase in pericardial effusion, but there was a new collection of fluid at the atrioventricular groove (Figure 3(a)), compressing on the left atrium. There was neither pulsus paradoxus on hemodynamic monitoring nor other echocardiographic features of cardiac tamponade. Doppler echocardiography showed an increased mitral inflow gradient of 8 mmHg, suggesting functional mitral stenosis (Figure 3(b)).

**Figure 4:**
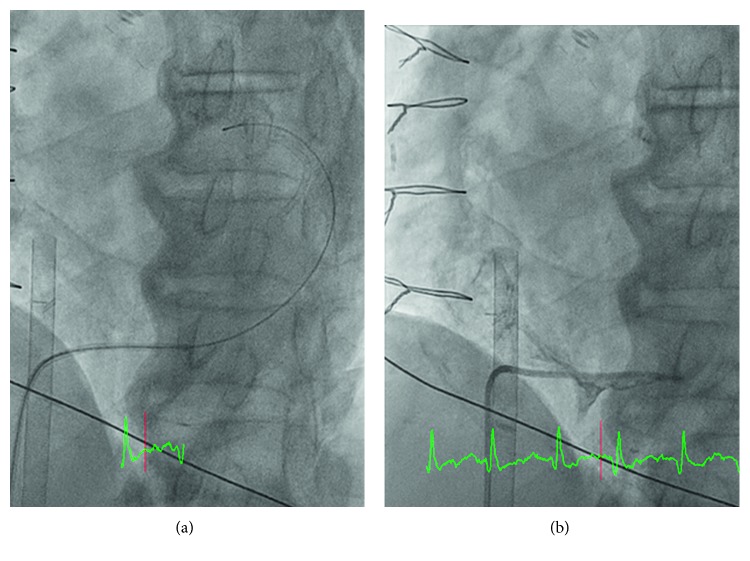
(a) Cannulation of the coronary sinus with an AL-1 catheter and hydrophilic wire. (b) Contrast injection through the AL-1 catheter showing compression and no flow of the coronary sinus.

Coronary sinus cannulation was attempted (Figure 4(a)), and the venogram showed no flow in coronary sinus (Figure 4(b)), suggesting extrinsic compression of the coronary sinus. Repeat pericardiocentesis was not attempted, as the fluid collection was posterior and there were no accessible windows.

**Figure 5:**
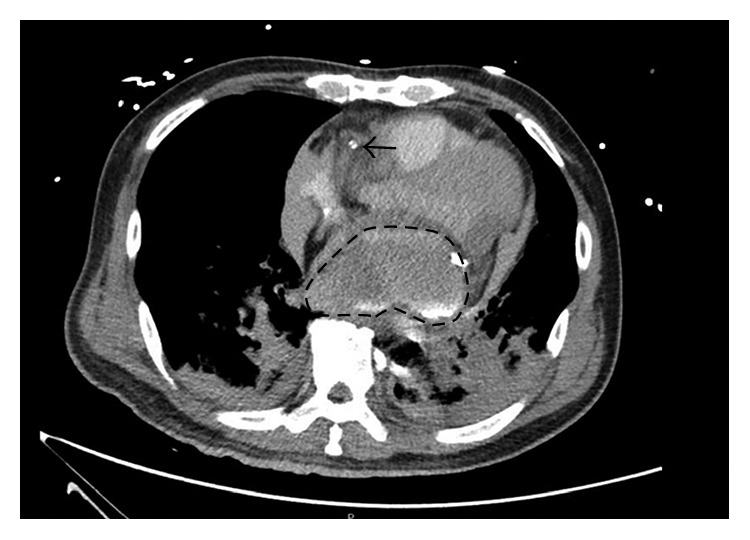
CT chest showing large hematoma arising from atrioventricular groove, with contrast from coronary angiogram, compressing on left atrium. The coronary sinus cannot be seen in the scan due to severe extrinsic compression. The arrow shows the ECMO cannula at the superior vena cava and right atrium junction.

An urgent computed tomography scan was performed confirming the presence of the large 7.6 cm × 3.9 cm collection in the posterior aspect of the mediastinum extrinsically compressing and displacing the left atrium superiorly and to the right (Figure 5). The coronary sinus could not be visualized due to extrinsic compression by the hematoma.

The patient was sent for emergent surgical evacuation of the hematoma with findings of large amounts of subepicardial hematoma, which had dissected the epicardium from the myocardium with left atrial compression. There were also multiple adhesions noted intraoperatively, likely from his previous cardiac bypass surgery. Hemodynamics improved after hemostasis and evacuation of clots compressing the left atrium and coronary sinus.

However, the patient deteriorated multiple times during the hospital stay due to reaccumulation of hematoma and had to undergo repeat sternotomies and evacuation of hematoma. His course was complicated by oliguric acute kidney injury requiring continuous renal replacement therapy, sepsis, and ischemic stroke. He was supported by VA ECMO throughout and died seven weeks after his procedure due to multiorgan failure.

## 2. Discussion

The incidence of coronary perforation is reported to be 0.3–0.6% of all PCI procedures in combined reviews of approximately 50,000 cases [1, 2]. The incidence is usually higher with the use of newer devices such as rotational artherectomy, direction artherectomy, cutting balloon, and excimer laser, and when PCI is performed in postcardiac bypass patients. Most lesions can be treated by temporizing with balloon inflation at the site of perforation for up to 15 minutes to stop the blood flow into the pericardium, emergency pericardiocentesis if there are signs of hemodynamic instability, followed by placement of embolization coils for distal perforations or polytetrafluoroethylene membrane-covered stents to seal off further flow through the perforation [3, 4]. However, if there is continued bleeding or hemodynamic instability, emergency referral must be made to cardiothoracic surgery for surgical repair.

Coronary sinus compression as a sign of cardiac tamponade secondary to increase pericardial pressure [5] and tamponade phenomena secondary to left atrial compression are rare and difficult to detect, with few case reports in literature [1, 3]. These cases mostly occur during intervention to the circumflex artery due to its position in relation to its surrounding structures.

The circumflex artery, which branches off from the left main artery, runs along the atrioventricular groove around the left atrium, giving rise to left marginal branches in the process. It continues around the left atrium and terminates in the posterior inferior aspect of the heart. The coronary sinus, which receives drainage from the great, middle, and posterior left ventricular veins, is a low-pressure venous conduit that runs along the atrioventricular groove, which is in close proximity to the left circumflex artery [5]. Posterior to the left atrium lays the oblique pericardial sinus, bounded by the inferior vena cava, right pulmonary veins, and the left pulmonary veins.

This case illustrates the anatomic and physiologic consequences during circumflex artery perforation. Blood collects in the oblique sinus, and as the left atrium is fixed to the parietal pericardium by the entry of the pulmonary veins, fluid in this fixed space accumulates pressure [6]. As the blood volume increases, pressure accumulates until it compresses on the low-pressure coronary sinus, obstructing the flow. Previous cardiac bypass surgery could have formed adhesions around the pericardium, preventing redistribution of blood around the pericardial space. As the coronary sinus provides the primary drainage of the myocardial venous system, abrupt occlusion is likely to result in significant elevation of the transcapillary pressure. This in turn is likely to result in reduced myocardial perfusion pressure, aggravating myocardial ischemia [7], explaining the patient's ischemic ECG changes and arrhythmic collapse. Further blood collection in the oblique sinus will tamponade the left atrium and obstruct left atrial filling. The stroke volume consequently decreases and cardiac output drops, causing hypotension.

There are few possible theories for the gradient across the mitral valve and functional mitral stenosis. Firstly, the hematoma externally compresses against the mitral annulus; this reduces the size of the mitral valve orifice and limits the movement of the annulus, affecting flow across the mitral valve. Secondly, the high external pressure from the hematoma obstructs filling of the atrium from the pulmonary veins; if the left ventricle function is relatively intact, this creates a suction force and gradient across the mitral valve.

Although extremely rare, it is important to recognize such subtle signs of tamponade following perforation during coronary angioplasty, especially in the circumflex artery and post-bypass surgery patients. This life-threatening complication requires clinical awareness for rapid diagnosis, and emergent referral needs to be made to cardiothoracic surgery for surgical treatment.
